# Simultaneous Resection for Colorectal and Liver Metastases, new equipment and personalized medicine

**DOI:** 10.7150/jca.90519

**Published:** 2024-01-01

**Authors:** Paul Zarogoulidis, Aris Ioannidis, Anastasios Vagionas, Eleni Isidora Perdikouri, Vagelis Christakidis, Marios Anemoulis, Isaak Kesisoglou, Dimitris Matthaios, Panagoula Oikonomou, Christina Nikolaou, Lavrentios Papalavrentios, Charalampos Charalampidis, Nikolaos Machairiotis, Vasilis Papadopoulos, Konstantinos Sapalidis

**Affiliations:** 1 3 rd Department of Surgery, ``AHEPA`` University Hospital, Aristotle University of Thessaloniki, Medical School, Thessaloniki, Greece.; 2 Surgery Department, General Clinic Private Hospital, Thessaloniki, Greece.; 3 Oncology Department, General Hospital of Kavala, Greece.; 4 Oncology Department, General Hospital of Volos, Greece.; 5 Oncology Department, General Hospital of Serres, Greece.; 6 Surgery Department, Genesis Private Clinic, Thessaloniki, Greece.; 7 Oncology Department, General Hospital of Rhodes, Rhodes, Greece.; 8 Second Department of Surgery, University Hospital of Alexandroupolis, Medical School, Democritus University of Thrace, Alexandroupolis, Greece.; 9 Gastroenterologist, Private Cabinet, Thessaloniki, Greece.; 10 Pathology Department, University of Cyprus, Cyprus.; 11 Third Department of Obstetrics and Gynecology, University General Hospital “ATTIKON”, Medical School of the National and Kapodistrian University of Athens, Athens, Greece.; 12 Oncology Department, University General Hospital of Larissa, University of Thessaly, Greece.

**Keywords:** colon cancer, liver metastasis, simultaneous liver metastasis surgery

## Abstract

Nowadays we perform synchronous colorectal cancer resection along with synchronous liver metastases. We investigated whether colon resection first is safer than liver resection first and if simultaneous surgeries are in general safe.

**Patients and Methods:** Twenty patients were included in our multicenter study. In our study patients had simultaneous laparoscopic resection of primary colorectal cancer and liver metastases. The patients included were divided into two groups based on their first surgery. Group A had colon resection first (n = 10) and group B had liver resection first (n = 10). All adverse effects and outcomes were compared after the first day of hospitalization.

**Results:** The only difference between the two groups was the operative blood loss. It was observed to be less in group B.

**Conclusion:** In our study we did not observe any significant difference regarding the order of the operation.

## Introduction

**Figure 1:**
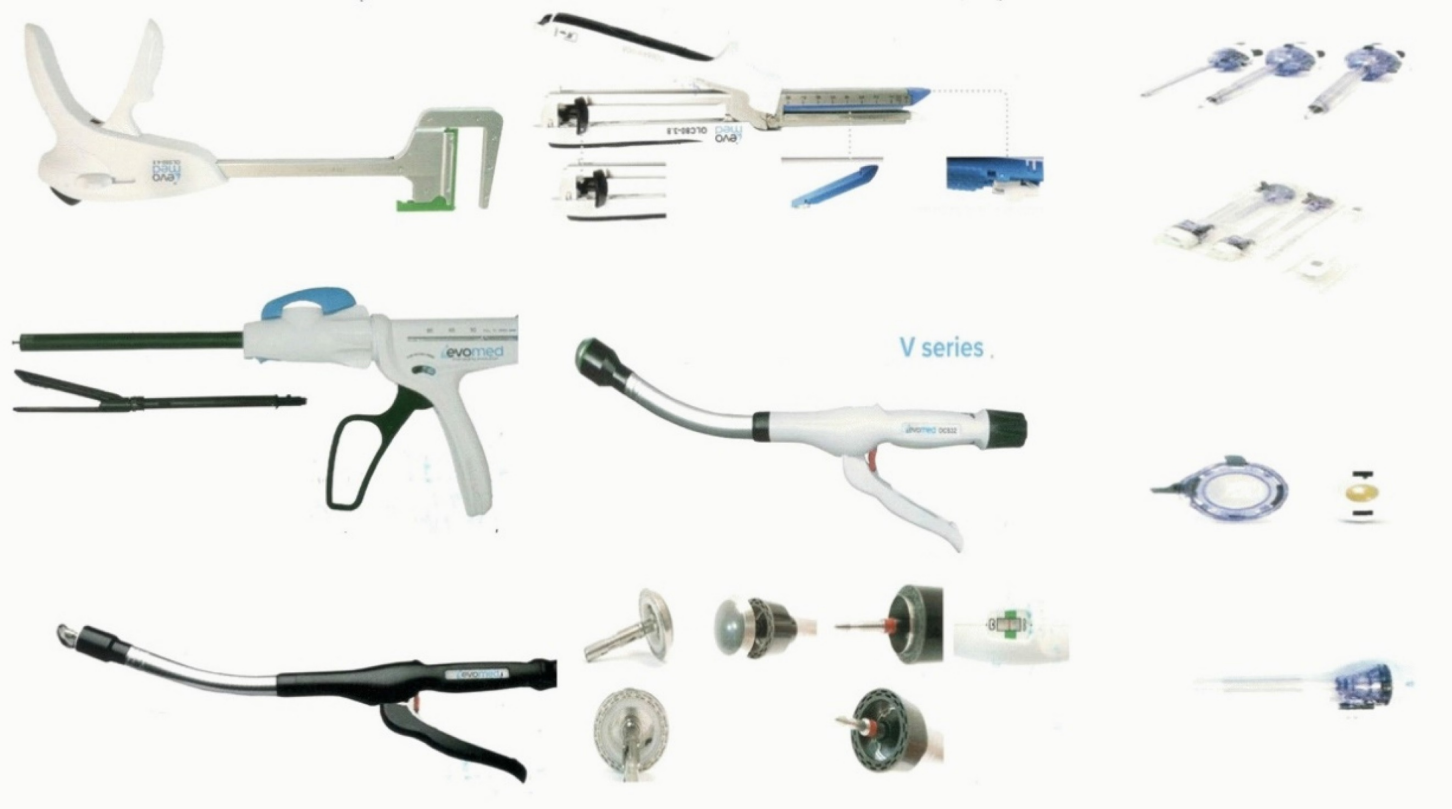
'Antisel' new laparoscopic equipment.

Colorectal cancer is a very common type of cancer. Colorectal liver metastases (CRLMs) are observed upon diagnosis in up to 50% of patients with colorectal 1, 2. Nowadays we have the diagnostic tools and treatment techniques to treat both liver metastases (SLMs) and colon cancer. Liver metastases is an independent prognostic factor for the survival of colon cancer patients 3-5. In the case of unresectable liver metastases then we can use chemotherapy along with local ablation techniques and then perform hepatectomy with excellent results 6, 7. It has been previously published that simultaneous resection of primary colon cancer and liver metastases is associated with similar oncological outcomes 8. In any case when unresectable liver matastasis are diagnosed chemotherapy is administered first along with local therapy when applicable. In the case were both colon cancer and liver metastases are resectable then silmutaneous surgical resection is an option. Simultaneous resection is possible in a single operation. Although it was considered until recently that simultaneous resection might increase postoperative complications, recent published data elucidated that postoperative complications rates were very low due to novel diagnostic and surgical equipment 9,10, and moreover the length of hospital stay and cost was lower 11 (Figure 1). Until recently the safety of simultaneous resection was unclear. Moreover; the best surgical order regarding complications (colon first or liver-first approach) was not been evaluated. Simultaneous laparoscopic resection of primary tumor and liver metastases is definitely feasible and it has been previously performed 12. Furthermore; previously published data reported fewer postoperative complications 12, 13. However; we still need more studies to elucidate the proper order in silmutaneous primary colon cancer with liver metastasis.

## Patients and Methods

In our multicenter retrospective study we recruited twenty patients who had simultaneous resection of primary colon cancer and liver metastasis. We divided the patients into two groups, group A and B based on the operative order. In group A; there was Colon-first operation and in group B liver-first operation. All clinical, operative and postoperative complications were recorded and compared. The study was approved by the investigational review board of 3rd Department of Surgery, ``AHEPA`` University Hospital, Aristotle University of Thessaloniki, Medical School, Thessaloniki, Greece study protocol (January 2020-March 2022) IRB approval number 12/2019 and the study was carried out in accordance with the Declaration of Helsinki.

We collected data regarding the: sex, age, body mass index (BMI), primary tumor site (right/left/rectal), primary tumor diameter, number of involved hepatic segments American Society of Anesthetists-physical status, number of metastatic liver lesions, largest diameter of liver metastases. Moreover; we recorded the presence or absence of preoperative chemotherapy, and chemotherapy regimen along with lobar surgical resection. The surgical approach was categorized as; right colon, left colon and rectal colon. The operative order was left to the surgeon's opinion.

## Results

Main characteristics are presented in Table I. In our study we had 10 patients in group A and 10 in group B. No significant differences were observed in demographics. The position of the primary colorectal cancer was right/left/rectum in 8/8/4. We observed in group B a significantly higher rates of rectal cancer compared to that of the group A. The mean primary tumor diameter was 39±13 mm.

In 11 patients we observed one silmutaneous liver matastasis (SLMs), in 7 patients there were two SLMs, and in 2 patients there were ≥2. The median number of hepatic segments involved were 2. Silmutaneous liver matastasis was unilobar in eleven patients, and bilobar for nine. The largest diameter of liver metastases was 19mm. In seven patients chemotherapy was administered preoperatably (Table 2).

However; there was no significant between the operative time, for the two groups. Postoperative complications were observed in eight patients (Table 3). Furthermore; no difference was observed for both groups regarding the nutrition status after surgery and postoperative hospital stay. No fatal event or adverse effect was observed within the next 30 days for patients in both groups.

## Discussion

We reevaluated in our department the clinical impact of laparoscopic simultaneous resection for SLMs with colon cancer. We observed again as in previous studies that the operative order had no effect on the perioperative outcome. In group B which was the liver-first approach we observed lower blood loss as in previous studies. No significant difference in the anastomotic leakage was observed between the two groups in our study. We believe that the main reason for less blood loss in group B are technical and anatomical reasons of the procedure during hepatectomy. A technical and anatomical aspect is the low central venous pressure. This anatomy provides easy control of the hepatic 14. Laparoscopic technique has improved in the past twelve years and there is less blood loss. There was no statistical difference between the two groups regarding the operative time. Although in group B the operating time was slightly less. Based on previously published studies the positional difference of the patient's body between primary resection and during the resection of metastatic lesions has an impact in the operating time. All centers included in our multicenter study used different positions such as; right upper limb elevation, and semi-lateral decubitus position and use of the intercostal ports where necessary 15. In laparoscopic colectomy we use the normal position such as a supine or lithotomy position and therefore the operating time is less.

In group B were hepatectomy is performed the rearrangement of the patient's position takes less time. We use the Pringle maneuver to control liver inflow 16-21. Although, sometimes lower volume may is observed which causes circulatory disorders in organs, and therefore performing the Pringle should be used only when necessary. In our study no significant difference in postoperative complication rates were observed for group A and B. We were not able to assess operative time and blood loss during colectomy and hepatectomy separately. Moreover; the long-term outcome has not been evaluated after 30 days. In previous studies when simultaneous laparoscopic resection for CRLM, was correlated with open surgery it the long-term outcomes observed were similar 22, 23. In these studies simultaneous and staged resection for CRLM did not provide any difference for 24 disease-free nor overall survival 25, 26. In our retrospective study there were significant differences in the number of liver metastases, surgical procedures for resection, the number of affected hepatic segments, and the number of patients were insufficient to compare two groups with similar backgrounds. We do not have any additional data whether local treatment plays any significant role in the disease free or survival rates. Major limitation of our study is the small number of patients, which was mostly attributed to the fear of the patients since simultaneous surgeries are not the gold standard in these situations. Moreover; we included in our study only patients that were fit to undergo such a surgery either in group A, or B. A larger study is needed to clarify several perioperative issues such as blood loss, or the technical experience that the operations much have.

## Conclusion

In conclusion the order simultaneous resection of primary and liver metastatic lesions, does not affect the short-term surgical outcomes and adverse effects. However; increased operative blood loss is observed in colon-first resections. Based on previous references-studies, our new equipment used, had similar results to the other studies, similar adverse effects and positive results with less than 5 days of hospitilisation.

## Figures and Tables

**Table I:** Demographic, clinical, and surgical characteristics of study patients

			APPROACH		
Demographic		Total (N=20)	Primary first (N=10)	Liver first (N=10)	p-Value
Age, years	Mean±SD	62±12	62±12	59±12	0.86
Gender, n	Male/female	12/8	3/7	2/6	0.16
BMI kg/m2	Mean±SD	20.2±3.6	24.7±2.1	20.5±2.3	0.12
ASA-PS, n (%)	1	11 (32.3%)	4 (8.1%)	7 (45%)	0.156
	2	7 (64.0%)	4 (61.8%)	3 (45%)	
	3	2 (2.7%)	2 (9.1%)	0	
Primary site, n (%)	Right	9	6	3	0.025
	Left	10	4	6	
	Rectum	1	0	1	
Primary tumor diameter, mm	Mean±SD	40±12	49±11	38±12	0.056
SLM, n (%)	Median no. (range)	2 (1-10)	2 (1-6)	2 (1-10)	0.85
	1 SLM	10	5	5	
	2 SLM	4	2	2	
	≥ 3 SLM	6	2	4	
Segments involved, n	Median (range)	2 (1-5)	1 (1-5)	2 (1-4)	
	Unilobar	10	5	5	0.53
	Bilobar	10	5	5	0.52
Largest LM diameter, mm	Mean±SD	20±18	19±14	22±21	0.20
Preoperative chemotherapy, n (%)	FOLFOX +mAb	2 (11.8%)	3 (16.2%)	1 (10.5%)	0.66
	SOX +mAb	0	0	0	
	FOLFIRI +mAb	1	1	0	
	XELOX +mAb	0	0	0	
	FOLFOXIRI +mAb	1	0	1	
	IRIS +mAb	1	0	1	
	FOLFOX +mAb	1	1	0	

**Table 2:** Operative methods.

	Total (N=20)	Primary first (N=10)	Liver first (N=10)
Primar Laparoscopic	20	10	10
Liver Laparoscopic	20	10	10
Hand-assist	5	5	0
Hybrid	6	4	2
Pure laparoscopic	20	2	10
Colectomy			
Ileocecal resection	3	2	1
Right hemicolectomy	5	3	2
Sigmoid colectomy	7	3	4
Left hemicolectomy	1	1	0
Partial colectomy	3	2	2
High anterior resection	2	0	2
Low anterior resection	4	0	4
Intersphincteric resection	2	0	2
Hepatectomy Anatomical resection	6	2	4
Parenchymal resection	17	7	10
Both	4	2	2

**Table 3:** Operative and postoperative results.

Parameter		Total (N=20)	Primary first (N=10)	Liver first (N=10)	p-Value
Operative time, min	Median (range)	560 (374-879)	542 (450-724)	593 (374-837)	0.21
Time in the operating room, min	Median (range)	686 (551-1,152)	841 (551-1,183)	731 (531-1,162)	0.24
Blood loss, ml	Median (range)	435 (5-2,066)	490 (94-2,086)	154 (7-670)	0.04
Complications, n	Surgical site infection				
	Abdominal abscess	1	1	0	
	Biloma	1	0	1	
	Biliary fistula	1	1	0	
	Anastomosis leakage	2	2	0	
	Paralytic ileus	0	0	0	
	Pneumothorax1	0	0	0	
	Pleuraleffusion/ascites	0	0	0	
	Pneumonia/urinary	1	0	1	
	Tract infection	0	0	0	
	None	20	6	5	
Clavien-Dindo classification, n (%)	≥II	6 (15.5%)	2 (24.2%)	1 (11.3%)	0.33
	≥III	4 (13.7%)	3 (25.2%)	1 (6.2%)	0.12
Start of oral intake, days	Median (range)	3 (2-6)	3(2-6)	4 (2-7)	0.24
Postoperative hospital stay, days	Median (range)	13 (10-50)	14 (10-50)	12 (10-25)	0.21
Re-operation within 30 days	Yes	0	0	0	
Death within 30 days	Yes	0	0	0	

## References

[1] Kemeny N (2010). The management of resectable and unresectable liver metastases from colorectal cancer. Current opinion in oncology.

[2] Araujo RLC, Figueiredo MN, Sanctis MA, Romagnolo LGC, Linhares MM, Melani AGF (2020). Decision making process in simultaneous laparoscopic resection of colorectal cancer and liver metastases. Review of literature. Acta cirurgica brasileira.

[3] Creasy JM, Sadot E, Koerkamp BG, Chou JF, Gonen M, Kemeny NE (2018). The Impact of Primary Tumor Location on Long-Term Survival in Patients Undergoing Hepatic Resection for Metastatic Colon Cancer. Annals of surgical oncology.

[4] Tomlinson JS, Jarnagin WR, DeMatteo RP, Fong Y, Kornprat P, Gonen M (2007). Actual 10-year survival after resection of colorectal liver metastases defines cure. Journal of clinical oncology: official journal of the American Society of Clinical Oncology.

[5] Fong Y, Fortner J, Sun RL, Brennan MF, Blumgart LH (1999). Clinical score for predicting recurrence after hepatic resection for metastatic colorectal cancer: analysis of 1001 consecutive cases. Annals of surgery.

[6] Beppu T, Miyamoto Y, Sakamoto Y, Imai K, Nitta H, Hayashi H (2014). Chemotherapy and targeted therapy for patients with initially unresectable colorectal liver metastases, focusing on conversion hepatectomy and long-term survival. Annals of surgical oncology.

[7] Hayashi H, Beppu T, Sakamoto Y, Miyamoto Y, Yokoyama N, Higashi T (2015). Prognostic value of Ki-67 expression in conversion therapy for colorectal liver-limited metastases. American journal of cancer research.

[8] Brouquet A, Mortenson MM, Vauthey JN, Rodriguez-Bigas MA, Overman MJ, Chang GJ (2010). Surgical strategies for synchronous colorectal liver metastases in 156 consecutive patients: classic, combined or reverse strategy?. Journal of the American College of Surgeons.

[9] Chen J, Li Q, Wang C, Zhu H, Shi Y, Zhao G (2011). Simultaneous vs. staged resection for synchronous colorectal liver metastases: a metaanalysis. International journal of colorectal disease.

[10] Boudjema K, Locher C, Sabbagh C, Ortega-Deballon P, Heyd B, Bachellier P (2021). Simultaneous Versus Delayed Resection for Initially Resectable Synchronous Colorectal Cancer Liver Metastases: A Prospective, Open-label, Randomized, Controlled Trial. Annals of surgery.

[11] Silberhumer GR, Paty PB, Temple LK, Araujo RL, Denton B, Gonen M (2015). Simultaneous resection for rectal cancer with synchronous liver metastasis is a safe procedure. American journal of surgery.

[12] Miyamoto Y, Beppu T, Sakamoto Y, Imai K, Hayashi H, Nitta H (2015). Simultaneous Laparoscopic Resection of Primary Tumor and Liver Metastases for Colorectal Cancer: Surgical Technique and Short-Term Outcome. Hepato-gastroenterology.

[13] Fretland AA, Sokolov A, Postriganova N, Kazaryan AM, Pischke SE, Nilsson PH (2015). Inflammatory Response After Laparoscopic Versus Open Resection of Colorectal Liver Metastases: Data From the Oslo-CoMet Trial. Medicine.

[14] Melendez JA, Arslan V, Fischer ME, Wuest D, Jarnagin WR, Fong Y (1998). Perioperative outcomes of major hepatic resections under low central venous pressure anesthesia: blood loss, blood transfusion, and the risk of postoperative renal dysfunction. Journal of the American College of Surgeons.

[15] Hayashi H, Yamashita YI, Okabe H, Imai K, Higashi T, Yamamura K (2020). Varied application of intercostal trans-diaphragmatic ports for laparoscopic hepatectomy. PloS one.

[16] Weiss MJ, Ito H, Araujo RL, Zabor EC, Gonen M, D'Angelica MI (2013). Hepatic pedicle clamping during hepatic resection for colorectal liver metastases: no impact on survival or hepatic recurrence. Annals of surgical oncology.

[17] Yaqub S, Margonis GA, Soreide K (2023). Staged or Simultaneous Surgery for Colon or Rectal Cancer with Synchronous Liver Metastases: Implications for Study Design and Clinical Endpoints. Cancers.

[18] Mankarious MM, Portolese AC, Hoskins MA, Deutsch MJ, Jeganathan NA, Scow JS (2023). Neoadjuvant chemotherapy does not increase risk for anastomotic leak for simultaneous resection of primary colon cancer with synchronous liver metastasis: A NSQIP-colectomy analysis. Journal of surgical oncology.

[19] Kazi M, Patkar S, Desouza A, Goel M, Saklani A (2023). Simultaneous laparoscopic complete mesocolic excision and liver metastasectomy for colorectal liver metastasis in difficult segments. Techniques in coloproctology.

[20] Hamzaoui Y, Genova P, Peschaud F, Malafosse R, El Hajjam M, Lupinacci RM (2022). Safeness of Simultaneous Colonic Resection and Hepatic Radiofrequency Ablation. JSLS: Journal of the Society of Laparoendoscopic Surgeons.

[21] Minciuna CE, Tudor S, Micu A, Diaconescu A, Alexandrescu ST, Vasilescu C (2022). Safety and Efficacy of Simultaneous Resection of Gastric Carcinoma and Synchronous Liver Metastasis-A Western Center Experience. Medicina.

[22] Takasu C, Shimada M, Sato H, Miyatani T, Imura S, Morine Y (2014). Benefits of simultaneous laparoscopic resection of primary colorectal cancer and liver metastases. Asian journal of endoscopic surgery.

[23] Tranchart H, Fuks D, Vigano L, Ferretti S, Paye F, Wakabayashi G (2016). Laparoscopic simultaneous resection of colorectal primary tumor and liver metastases: a propensity score matching analysis. Surgical endoscopy.

[24] Deng Y, Chen Q, Chen J, Zhang Y, Zhao J, Bi X (2023). An elevated preoperative cholesterol-to-lymphocyte ratio predicts unfavourable outcomes in colorectal cancer liver metastasis patients receiving simultaneous resections: a retrospective study. BMC surgery.

[25] Silberhumer GR, Paty PB, Denton B, Guillem J, Gonen M, Araujo RLC (2016). Long-term oncologic outcomes for simultaneous resection of synchronous metastatic liver and primary colorectal cancer. Surgery.

[26] Kim NR, Alhothaifi ED, Han DH, Choi JS, Choi GH (2023). Prognostic impact of R1 resection margin in synchronous and simultaneous colorectal liver metastasis resection: a retrospective cohort study. World journal of surgical oncology.

